
JOURNAL INFORMATION
==============================
NLM Title Abbreviation: Neth Heart J
Journal ID: Neth Heart J
NLM Journal ID: 101095458

Netherlands Heart Journal

ISSN: 1568-5888
EISSN: 1876-6250
Publisher: Springer

ARTICLE INFORMATION
==============================
PMCID: PMC6311155
DOI: 10.1007/s12471-018-1220-3
Article ID: 1220
Article version: 1
Subjects: Thanks to Reviewers

Thanks to reviewers

Electronic publication date: 2018 Dec 11
Print publication date: 2019 Jan
Volume: 27
Issue: 1
First page: 58
Last page: 59
Copyright: © The Author(s) 2018
License: Open Access This article is distributed under the terms of the Creative Commons Attribution 4.0 International License (http://creativecommons.org/licenses/by/4.0/), which permits unrestricted use, distribution, and reproduction in any medium, provided you give appropriate credit to the original author(s) and the source, provide a link to the Creative Commons license, and indicate if changes were made.
License URL: https://creativecommons.org/licenses/by/4.0/

issue-copyright-statement: © The Author(s) 2019

==============================
Neth Heart J (2019) 27:58–59
https://doi.org/10.1007/s12471-018-1220-3

### Thanks to reviewers

Published online: 11 December 2018
© The Author(s) 2018

The Editorial Team would like to extend its gratitude to all reviewers for their time and advice which helped us to assess and improve the papers that were submitted to us in 2018. The reviewers' critical and constructive feedback greatly helped to raise the journal's quality. We would like to thank Sing-Chien Yap (Erasmus MC, Rotterdam) who performed the most reviews in 2018.

Wim Aengevaeren
Marco Alings
Reza Alizadeh Dehnavi
Giovanni Amoroso
Elena Arbelo
Lorenzo Azzalini
Barbara Backus
Jippe Balt
Marcel Beijk
Sebastiaan Bekkers
Rypko Beukema
Geertruida Bijvoet
Yuri Blaauw
Eric Boersma
Lucas Boersma
Bas Boukens
Richard Braam
Sander Bramer
Freerk Broeyer
Marc Brouwer
Cyril Camaro
Steven Chamuleau
Jin Cheng
Imke Christiaans
Bimmer Claessen
Kevin Damman
Peter Damman
Giuseppe D'Ancona
Menko de Boer
Peter de Jaegere
Jonas de Jong
Nicolaas De Jonge
Robbert de Winter
Admir Dedic
Ronak Delewi
Peter den Heijer
Hester den Ruijter
Dennis den Uijl
Dirk Donker
Dennis Dooijes
Anthonie Duijnhouwer
Suzette Elias-Smale
Howaida El-Said
Arif Elvan
Mireille Emans
Reinder Evertz
Sofie Gernaat
Erik Grove
Rainer Hambrecht
Magdalena Harakalova
Richard Hauer
Apollonia Helderman-van den Enden
Martin Hemels
José Henriques
Rik Hermanides
Alexander Hirsch
Niels Hoedemaker
Jeannette Hofmeijer
Patrick Houthuizen
Stef Hubbers
Kurt Huber
Michiel Hulleman
Alexander IJsselmuiden
Veronica Janssen
Luc Jordaens
Wouter Jukema
Bas Kietselaer
Paul Knaapen
W. Kok
Regina Konst
Dave Koolbergen
Stefan Koudstaal
Adriaan Kraaijeveld
Jos Kraal
Herbert Kroon
Narendra kumar
Andrew Landstrom
Jorrit Lemkes
Gerard Linssen
Roberto Lorusso
Caroline Lucas
Justin Luermans
Joost Lumens
Angela Maas
Alexander Maass
Olivier Manintveld
Matthijs Meijs
Casper Mihl
Mariagrazia Modena
Mohamed Mouden
Franz Neumann
Luc Noyez
Rutger Jan Nuis
Marish Oerlemans
Nicole Panhuyzen-Goedkoop
Ron Peters
Yigal Pinto
Laurent Pison
Nikki Pluymaekers
Marco Post
Pieter Postema
Saman Rasoul

Benno Rensing
Danielle Robbers-Visser
Tomas Robyns
Frans Rutten
Peyman Sardari Nia
Jeroen Schaap
Klaske Siegersma
Krischan Sjauw
Joep Smeets
Ruud Spee
Pieter Stella
Jurriën ten Berg
Tim ten Cate
Nienke Ter Hoeve
Arco Teske
Joep Thijssen
Ruben Tijssen
Leo Timmers
Pim Tonino
Serge Trines
Alicia Uijl
Alexander Van de Bruaene
Tim van de Hoef
Maarten van den Berg
R.B.A. van den Brink
Joep van der Harst
Jeroen van der Heijden
Joost van der Heijden
Nina van der Hoeven
Pieter van der Vleuten
Christian van der Werf
Arie van dijk
Vincent van Dijk
S. Van Doorn
Vanessa van Empel
Isabelle van Gelder
Linda van Laake
Kicky van Leeuwen
Petra van Pol
Philippe van Rosendael
Arnoud van 't Hof
G. Veen
Freek Verheugt
Marije Vis
Pieter Vlaar
J.H. von der Thusen
Alexander Vonk
Michiel Voskuil
Tom Vromen
Adrianus Wijnmaalen
Thierry Wildbergh
Arthur Wilde
Bo Winkel
Janneke Wittekoek
Jochen Wöhrle
Sing-Chien Yap
Dilek Yilmaz
Majd Zreik
Bastiaan Zwart
Koos Zwinderman
